# Prognostic Indicators of Non-Transection Nerve Injury and Vocal Fold Motion Impairment After Thyroid Surgery – Correlation Between Intraoperative Neuromonitoring Findings and Perioperative Voice Parameters

**DOI:** 10.3389/fendo.2021.755231

**Published:** 2021-11-30

**Authors:** Tzu-Yen Huang, Wing-Hei Viola Yu, Feng-Yu Chiang, Che-Wei Wu, Shih-Chen Fu, An-Shun Tai, Yi-Chu Lin, Hsin-Yi Tseng, Ka-Wo Lee, Sheng-Hsuan Lin

**Affiliations:** ^1^ International Thyroid Surgery Center, Department of Otolaryngology-Head and Neck Surgery, Kaohsiung Medical University Hospital, Faculty of Medicine, College of Medicine, Kaohsiung Medical University, Kaohsiung, Taiwan; ^2^ Department of Biological Science and Technology, National Yang Ming Chiao Tung University, Hsinchu, Taiwan; ^3^ Department of Biological Science and Technology, National Chiao Tung University, Hsinchu, Taiwan; ^4^ Department of Otolaryngology-Head and Neck Surgery, E-Da Hospital, Kaohsiung, Taiwan; ^5^ School of Medicine, College of Medicine, I-Shou University, Kaohsiung, Taiwan; ^6^ Department of Otolaryngology-Head and Neck Surgery, Kaohsiung Municipal Siaogang Hospital, Kaohsiung Medical University Hospital, Faculty of Medicine, College of Medicine, Kaohsiung Medical University, Kaohsiung, Taiwan; ^7^ Institute of Statistics, National Yang Ming Chiao Tung University, Hsinchu, Taiwan; ^8^ Institute of Statistics, National Chiao Tung University, Hsinchu, Taiwan; ^9^ Department of Otolaryngology-Head and Neck Surgery, Kaohsiung Municipal Tatung Hospital, Kaohsiung Medical University Hospital, Faculty of Medicine, College of Medicine, Kaohsiung Medical University, Kaohsiung, Taiwan; ^10^ Institute of Data Science and Engineering, National Yang Ming Chiao Tung University, Hsinchu, Taiwan; ^11^ Institute of Data Science and Engineering, National Chiao Tung University, Hsinchu, Taiwan

**Keywords:** thyroid surgery, intraoperative neuromonitoring (IONM), vocal fold motion, recurrent laryngeal nerve (RLN), index of voice and swallowing handicap of thyroidectomy (IVST), subjective/objective voice analysis

## Abstract

**Objectives:**

In patients with recurrent laryngeal nerve (RLN) injury after thyroid surgery, unrecovered vocal fold motion (VFM) and subjective voice impairment cause extreme distress. For surgeons, treating these poor outcomes is extremely challenging. To enable early treatment of VFM impairment, this study evaluated prognostic indicators of non-transection RLN injury and VFM impairment after thyroid surgery and evaluated correlations between intraoperative neuromonitoring (IONM) findings and perioperative voice parameters.

**Methods:**

82 adult patients had postoperative VFM impairment after thyroidectomy were enrolled. Demographic characteristics, RLN electromyography (EMG), and RLN injury mechanism were compared. Multi-dimensional voice program, voice range profile and Index of voice and swallowing handicap of thyroidectomy (IVST) were administered during I-preoperative; II-immediate, III-short-term and IV-long-term postoperative periods. The patients were divided into R/U Group according to the VFM was recovered/unrecovered 3 months after surgery. The patients in U Group were divided into U1/U2 Group according to total IVST score change was <4 and ≥4 during period-IV.

**Results:**

Compared to R Group (42 patients), U Group (38 patients) had significantly more patients with EMG >90% decrease in the injured RLN (p<0.001) and thermal injury as the RLN injury mechanism (p=0.002). Voice parameter impairments were more severe in U Group compared to R Group. Compared to U1 group (19 patients), U2 Group (19 patients) had a significantly larger proportion of patients with EMG decrease >90% in the injured RLN (p=0.022) and thermal injury as the RLN injury mechanism (p=0.017). A large pitch range decrease in period-II was a prognostic indicator of a moderate/severe long-term postoperative subjective voice impairment.

**Conclusion:**

This study is the first to evaluate correlations between IONM findings and voice outcomes in patients with VFM impairment after thyroid surgery. Thyroid surgeons should make every effort to avoid severe type RLN injury (e.g., thermal injury or injury causing EMG decrease >90%), which raises the risk of unrecovered VFM and moderate/severe long-term postoperative subjective voice impairment. Using objective voice parameters (e.g., pitch range) as prognostic indicators not only enables surgeons to earlier identify patients with low voice satisfaction after surgery, and also enable implementation of interventions sufficiently early to maintain quality of life.

## Introduction

Recurrent laryngeal nerve (RLN) injury during thyroid surgery is the most common etiology of vocal fold motion (VFM) impairment (1) and morbidity after this procedure and a leading cause of medico-legal litigation after thyroid surgery (2). To avoid RLN injury during thyroid surgery, adjunct use of intraoperative neuromonitoring (IONM) has gained widespread use for identifying the RLN early in thyroid surgery and for elucidating nerve injury mechanism (3–6). The incidence of RLN paralysis may be underestimated if VFM is not routinely examined (7). Previous works report that permanent RLN palsy occurs in 1–3% of thyroid surgeries and that temporary RLN palsy occurs in 1.4-38% of these procedures (8, 9). For comprehensive evaluation of RLN function before and after thyroid surgery, current practice guidelines recommend performing pre- and post-operative laryngofiberscopy routinely (6, 10).

Patients who had a RLN injury during thyroid surgery are expected to have VFM impairment after surgery and may also experience severe dysphonia, dyspnea, and aspiration (11). In patients with temporary RLN palsy caused by a non-transection RLN injury, Chiang et al. (12) reported that VFM usually recovers within 3 days to 4 months (mean, 30.7 days) after surgery. However, the course of VFM recovery depends on the nerve injury mechanism (13). Thermal injuries are associated with a long recovery time and severe histological disturbance in the endoneurium whereas mechanical injuries are associated with distorted epineuria and perineuria (13, 14). After RLN injury, natural neuromuscular compensation mechanisms may yield outcomes that are acceptable to the patient, including RLN function outcomes and voice outcomes (15). However, early assessment of voice performance is important for deciding subsequent voice intervention (16). There is evidence that voice interventions 3 months after RLN injury can greatly improve voice outcome after thyroid surgery (10, 17, 18).

Impaired VFM after thyroid surgery is considered evidence of RLN injury. Unrecovered VFM and subjective voice impairment degrade quality of life and satisfaction with treatment, which causes extreme distress in patients. For surgeons, treating these poor outcomes is also extremely challenging. To enable early identification and treatment of VFM impairment and to improve patient satisfaction with surgery outcomes, this study evaluated prognostic indicators of non-transection RLN injury and VFM impairment after thyroid surgery. Correlations between IONM findings and perioperative voice parameters were also investigated.

## Materials and Methods

**Figure 1:**
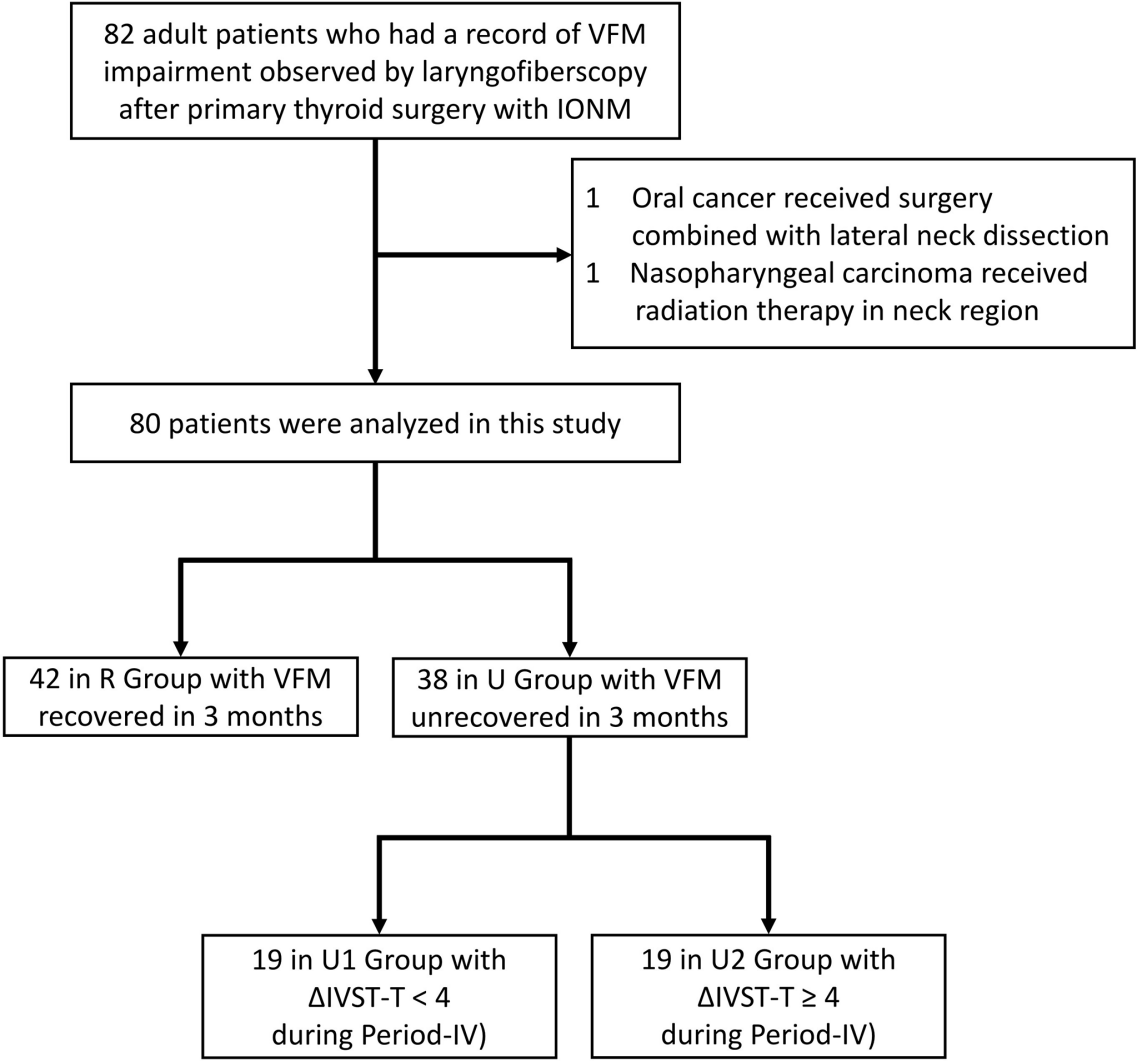
Flow diagram for inclusion and exclusion of patients. VFM, vocal fold motion; IONM, intraoperative neuromonitoring; IVST, Index of Voice and Swallowing Handicap of Thyroidectomy.

The subjects of this study were adult patients who had received IONM-assisted primary thyroid surgery by “IONM team” at a single institution (Kaohsiung Medical University Hospital, Taiwan) from June, 2013, to June, 2019. All patients had normal and symmetric VFM before surgery and had VFM impairment within 2 weeks after surgery. The total number of surgeries performed in this period was 1033, and the number of surgeries without VFM impairment was 951. The analysis included 82 (7.9%) patients after exclusion of one oral cancer patient who had received cancer surgery combined with lateral neck dissection and one nasopharyngeal carcinoma patient who had received radiation therapy in the neck region. The flowchart in **Figure 1** depicts the procedure for inclusion and exclusion of patients in this study. Ethical approval of this study was obtained from the Kaohsiung Medical University Hospital Institutional Review Board (KMUHIRB-E(I)-20200359). In all patients, vagus nerve function and RLN function were routinely evaluated using the standard four-step vagus nerve and RLN stimulation (V1-R1-R2-V2) procedure under IONM and the evoked electromyography (EMG) amplitudes were obtained and recorded (3, 19). The RLN EMG decrease was defined and calculated as the decrease in the EMG amplitude of the post-dissection R2 signal from the EMG amplitude of the pre-dissection R1 signal. No patient who had received bilateral thyroidectomy in this study had RLN signal >50% decreases on both sides. All exposed RLNs, including injury mechanisms (transection, mechanical, or thermal), were documented by photograph. Mechanical injury was classified as type 1 (segmental injury) or type 2 (global injury) according to the criteria proposed by Chiang et al. (3) and the INMSG (6). All patients received surgery by “IOINM team” routinely received a VFM survey. Laryngofiberscopy was documented by video in all patients before surgery and within 2 weeks after surgery. All patients with VFM impairment 2 weeks after surgery underwent additional monthly examinations until VFM recovery. Patients were divided into two groups according to VFM recovery: a recovered VFM group (R Group) and an unrecovered VFM group (U Group). The R Group criterion was laryngofiberscopic evidence of VFM impairment recovery within 3 months after thyroidectomy; the U group criterion was persistent VFM impairment more than 3 months after thyroidectomy. Patient information, including gender, age, surgery type (unilateral versus bilateral), and pathology results (benign versus malignant) was recorded and compared between groups.

### Objective and Subjective Voice Analysis

All patients underwent both subjective and objective voice analyses in four periods: period-I (preoperative period, within 2 months before surgery), period-II (immediate postoperative period, median duration of 3 days, range of 1-7 days), period-III (short-term postoperative period, median duration of 12 days, range of 7-30 days), and period-IV (long-term postoperative period, median duration of 40 days, range of 30-90 days).

All objective voice analyses in all subjects were performed by a single experienced speech-language pathologist (WHV. Y) using the Multidimensional Voice Program (model 5105, version 3.1.7; KayPENTAX, USA) and the Voice Range Profile (model 4326, version 3.3.0; KayPENTAX, USA). Multidimensional Voice Program analyses included mean fundamental frequency (mean F0), jitter, shimmer and noise-to-harmonic ratio whereas Voice Range Profile analyses included maximum pitch frequency (Fmax), minimum pitch frequency (Fmin), and pitch range (PR). The PR was defined as the number of semitones between Fmax and Fmin.

All preoperative and postoperative subjective voice analyses were performed using the 10-item Index of Voice and Swallowing Handicap of Thyroidectomy (IVST) (**Supplemental Table 1**), which provides an index of the main symptoms observed in the patient. Each item in this subjective assessment is scored from 0-2 (never, sometimes, and always), respectively. The two domains of the IVST are the voice domain (IVST-V; items 1-7; score range 0-14) and the swallowing domain (IVST-S; items 8-10; score range 0-6). Thus, the score range for the total IVST (IVST-T) is 0 to 20.

The equation for calculating postoperative change in objective voice analysis data was Δ= (B - A)/A, and the equation for calculating postoperative change in subjective voice analysis data was Δ= B – A, where A and B are preoperative and postoperative values, respectively.

Long-term postoperative subjective voice impairment was also evaluated in patients with unrecovered VFM (U Group). The U group was divided into patients with no/mild subjective voice impairment (ΔIVST-T < 4) in Period-IV (U1 Group) and patients with moderate/severe subjective voice impairment (ΔIVST-T ≥ 4) in Period-IV (U2 Group).

### Statistical Analysis

To analyze the variables, independent t test and Pearson chi-square test were performed using R software (version-3.4). A two-tailed p value less than 0.05 was considered statistically significant.

## Results

### Demographic Characteristics of R Group and U Group

**Table 1** compares the 42 (52.5%) patients in R Group and the 38 (47.5%) patients in U Group. Age, gender, surgical extent, and pathology results did not significantly differ between groups. The proportions of patients with <50%, 50-90%, >90% EMG decreases in the injured RLN were 0.0%, 83.3%, and 16.7% in R Group, respectively; the corresponding proportions in U Group were 0.0%, 44.7%, and 55.3%, respectively. That is, the proportions of patients with >90% EMG decreases were significantly larger in U Group compared to R Group (p<0.001).

**Table 1:** Demographic characteristics of Recovered VFM Group (R Group) and Unrecovered VFM Group (U Group).

	R Group	U Group	p value
Number (%)	42 (52.5%)	38 (47.5%)	
Age (mean ± SD)	50.7 ± 13.7	52.7 ± 12.4	0.497
Gender			0.073
Male (%)	11 (26.2%)	5 (13.2%)	
Female (%)	31 (73.8%)	33 (86.8%)	
Surgical extent			0.086
Unilateral (%)	7 (16.7%)	1 (2.6%)	
Bilateral (%)	35 (83.3%)	37 (97.4%)	
Pathology			0.069
Benign (%)	24 (57.1%)	14 (36.8%)	
Malignant (%)	18 (42.9%)	24 (73.2%)	
EMG decrease in the injured RLN			<0.001
<50% decreased (%)	0 (0.0%)	0 (0.0%)	
50-90% decreased (%)	35 (83.3%)	17 (44.7%)	
>90% decreased(%)	7 (16.7%)	21 (55.3%)	
RLN Injury mechanism and type			0.002
Transection (%)	0 (0.0%)	0 (0.0%)	
Mechanical (%)	42 (100.0%)	30 (78.9%)	
Type 1	33	27	
Type 2	9	3	
Thermal (%)	0 (0.0%)	8 (21.1%)	
Subjective voice impairment during period-IV			0.028*
No/Mild (ΔIVST-T <4) (%)	31 (73.8%)	19 (50.0%)	
Moderate/Severe (ΔIVST-T ≥4) (%)	11 (26.2%)	19 (50.0%)	
RLN reinnervation or nerve grafting	0 (0.0%)	0 (0.0%)	

VFM, vocal fold motion; SD, standard deviation; EMG, electromyography; RLN, recurrent laryngeal nerve; IVST, Index of Voice and Swallowing Handicap of Thyroidectomy; IVST-T, Total IVST score; period IV, Long-term postoperative period (range of 30-90 days).

*p value <0.05, showed significant difference.

No patients had transection injury in either the R Group or the U Group. All 42 (100.0%) patients in the R Group had mechanical injury (type 1 in 33 patients and type 2 in 9 patients). Among the 38 patients in U Group, 30 (78.9%) patients had mechanical injury, 27 had type 1 injury, 3 had type 2 injury; and 8 (21.1%) had thermal injury. The percentage of patients with thermal injury was significantly higher in U Group compared to R Group (p=0.002). The proportions of patients with no/mild (ΔIVST-T <4) and moderate/severe (ΔIVST-T ≥4) subjective voice impairment in period-IV were 73.8% and 26.2% in R Group, respectively, versus 50.0% and 50.0% in U Group, respectively. In period-IV, U group also had significantly more patients with moderate/severe subjective voice impairment compared to R Group (p=0.028). No patients in this study had received RLN reinnervation or nerve grafting.

### Voice Parameter Changes (Δ) Associated With Different VFM Outcomes

In **Supplemental Table 2**, the detailed voice parameters, voice parameter changes (Δ), and p values are compared between R Group and U Group in each follow-up period. Voice parameter comparisons between the two groups revealed significant differences in preoperative Fmax and preoperative PR. Postoperative parameters did not significantly differ. To minimize the influence of preoperative differences, voice parameter changes (Δ) were calculated and compared. In period-IV the U group had a ΔIVST-T score of 7.1 ± 6.8.

**Figure 2:**
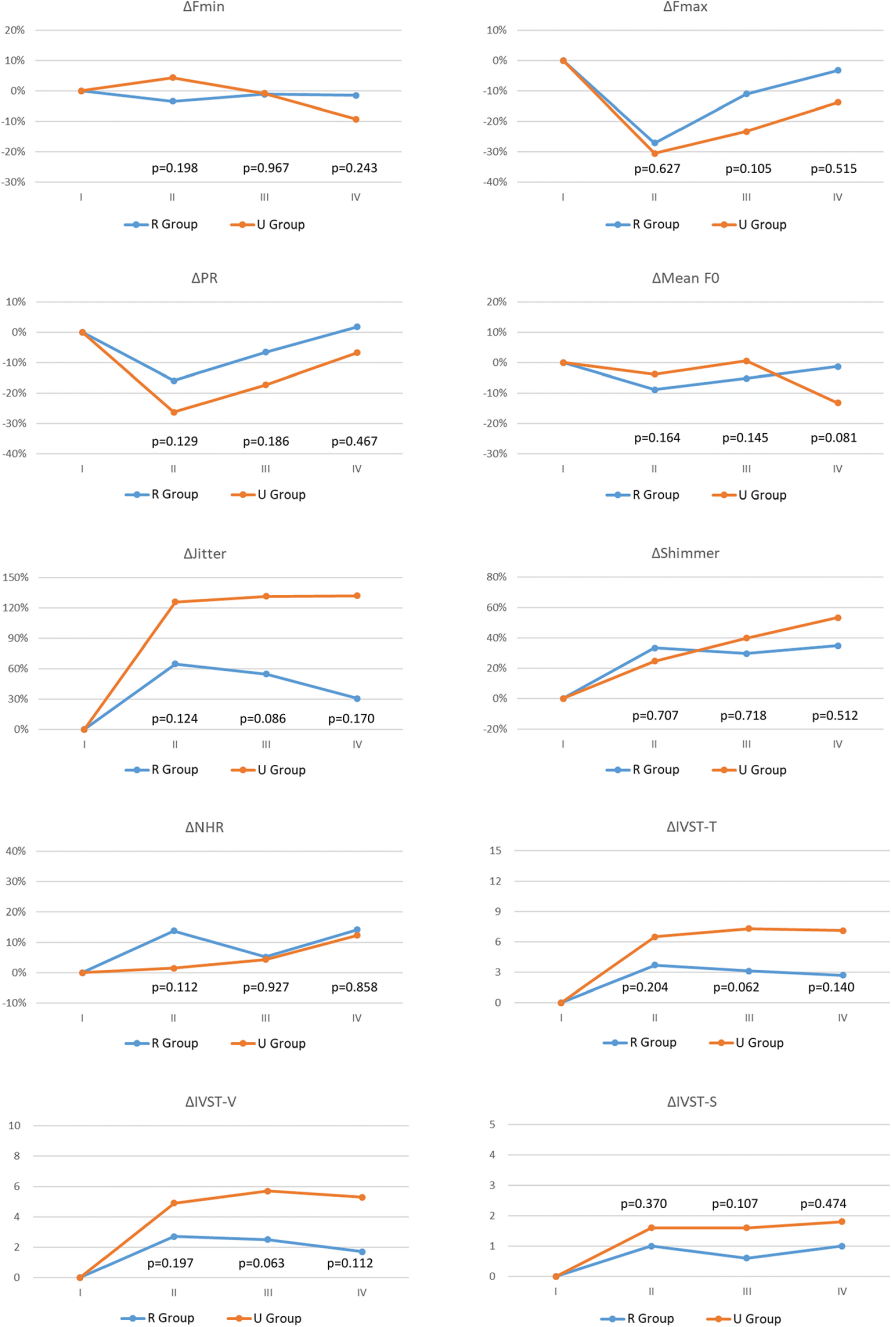
Voice parameter changes (Δ) with different vocal cord movement (VFM) outcomes: R Group (Blue line) and U Group (Red line). Fmax, Maximum pitch frequency; Fmin, Minimum pitch frequency; PR, Pitch range; Mean F0, mean fundamental frequency; NHR, noise-to-harmonic ratio; IVST, Index of Voice and Swallowing Handicap of Thyroidectomy; IVST-T, Total IVST score; IVST-V, IVST score of voice domain score; IVST-S, IVST score of swallowing domain. period I, Preoperative period (within 2 months before surgery); period II, Immediate postoperative period (median duration of 3 days; range of 1-7 days); period III, Short-term postoperative period (median duration of 12 days; range of7-30 days); period IV, Long-term postoperative period (median duration of 40 days, range of 30-90 days). The equation for calculating postoperative change in objective voice analysis (Fmax, Fmin, PR, Mean F0, Jitter, Shimmer, NHR) data was Δ= (B - A)/A, the unit is %; the equation for calculating postoperative change in subjective voice analysis (IVST-T, IVST-V, IVST-S) data was Δ= B – A, the unit is score. Where A and B are preoperative and postoperative values, respectively. The preoperative Δ is 0 in all the voice parameter. p value <0.05, showed significant difference.

**Figure 2** compares voice parameter changes (Δ) between the R Group and the U Group in each follow-up period. Due to large standard deviations, the two groups did not significantly differ in objective or subjective voice parameters in all follow-up periods. However, U Group showed larger impairments of voice parameters compared to R Group, especially in ΔFmax, ΔPR, ΔJitter, ΔIVST-T, ΔIVST-V, and ΔIVST-S.

### Demographic Characteristics and Objective Voice Parameter Changes (Δ) in U Group According to Severity of Subjective Voice Impairment in Long-Term Postoperative Period

**Figure 3:**
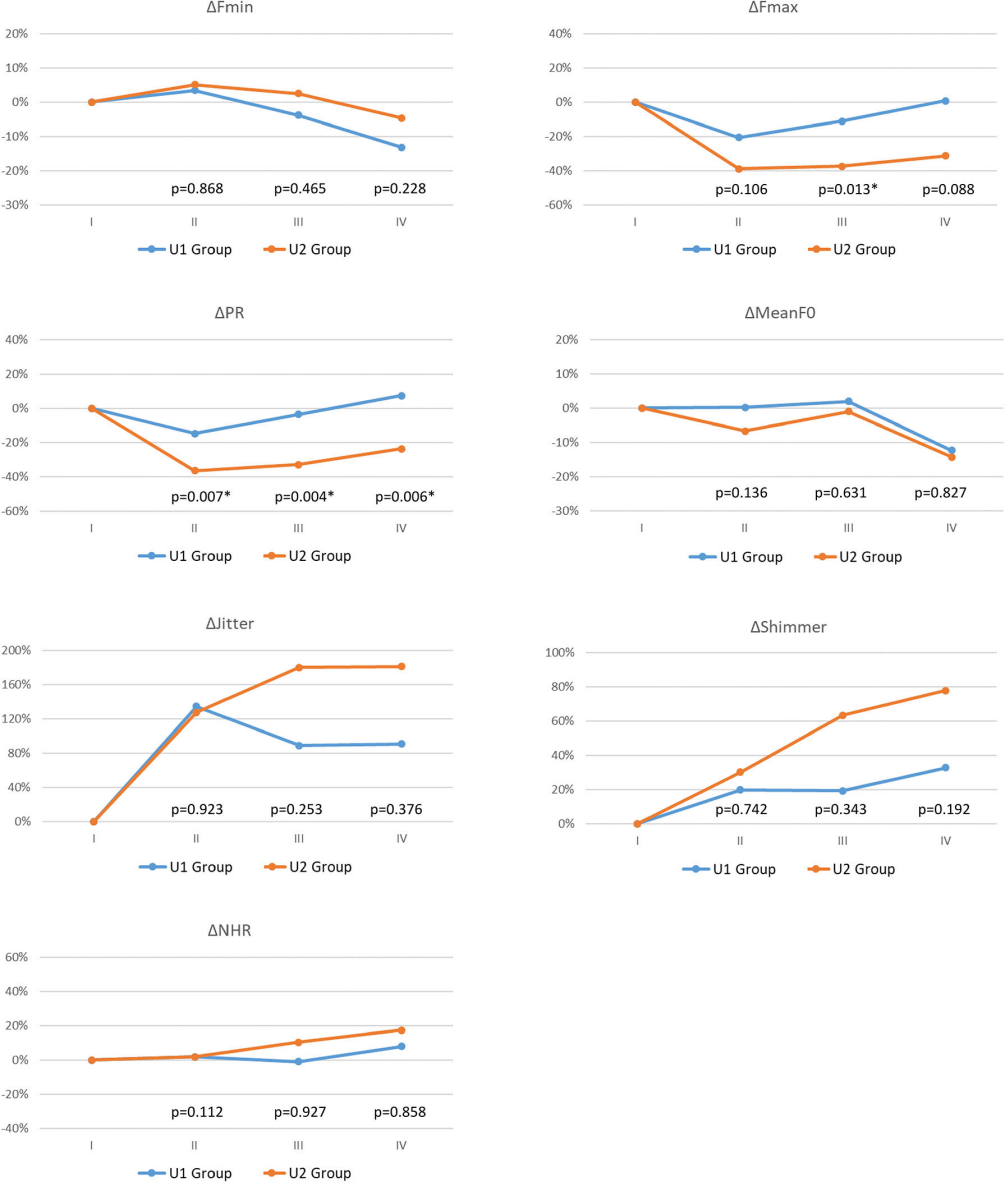
Objective voice parameter changes (Δ) with different subjective voice outcomes during long-term postoperative period (Period-IV): U1 Group (Blue line, ΔIVST-T< 4 during Period-IV) and U2 Group (Red line, ΔIVST-T ≥ 4 during Period-IV). The abbreviations of objective/subjective voice parameters, the definition of follow-up periods (I/II/III/IV), and the equation for calculating postoperative change in objective voice analysis are same in **Figure 2**. The preoperative Δ is 0 in all the voice parameter. p value <0.05, showed significant difference.

To evaluate the wide range of subjective voice impairment within U Group, **Table 2** shows the demographic characteristics of the U1 Group (no/mild subjective voice impairment) and U2 Group (moderate/severe subjective voice impairment). Compared to the U1 Group the U2 Group had significantly larger proportions of patients with EMG decrease >90% in the injured RLN (p=0.022) and patients with thermal injury as the primary RLN injury mechanism (p=0.017). **Figure 3** compares objective voice parameter changes (Δ) in the U1 Group and U2 Group in each follow-up period. In all postoperative periods, impairments in objective voice parameters were much larger in U2 Group compared to the U1 group. In period-II, ΔPR significantly p=0.007) differed between the U1 and U2 groups. In period-III, ΔFmax (p=0.013) and ΔPR (p=0.004) differed between the U1 and U2 groups; ΔJitter and ΔShimmer were larger in U2 Group compared to U1 Group. In period-IV, ΔPR (p=0.006) also significantly differed between groups; ΔFmax, ΔJitter, and ΔShimmer were larger in U2 Group compared to U1 Group.

**Table 2:** Demographic characteristic of patients in U Group with different subjective voice outcomes during long-term postoperative period (Period-IV).

	U1 Group (ΔIVST-T < 4 during Period-IV)	U2 Group (ΔIVST-T ≥ 4 during Period-IV)	p value
Case number	19 patients (50.0%)	19 patients (50.0%)	
Age	50.5 ± 10.9	54.9 ± 11.8	0.240
Gender			0.150
Male (%)	4 (21.1%)	1 (5.3%)	
Female (%)	15 (78.8%)	18 (94.7%)	
Surgical extent			0.311
Unilateral (%)	1 (5.3%)	0 (0.0%)	
Bilateral (%)	18 (94.7%)	19 (100.0%)	
Pathology			0.179
Benign (%)	5 (26.3%)	9 (47.4%)	
Malignant (%)	14 (73.7%)	10 (52.6%)	
EMG decrease in the injured RLN			0.022*
50-90% decreased (%)	12 (63.2%)	5 (26.3%)	
>90% decreased (%)	7 (36.8%)	14 (73.7%)	
RLN injury mechanism			0.017*
Mechanical (%)	18 (94.7%)	12 (63.2%)	
Thermal (%)	1 (5.3%)	7 (36.8%)	

IVST, Index of Voice and Swallowing Handicap of Thyroidectomy; IVST-T, Total IVST score; EMG, electromyography; RLN, recurrent laryngeal nerve.

Period IV (range of 30-90 days).

*p value <0.05, showed significant difference.

## Discussion

This study is the first to investigate correlations between IONM findings and voice outcomes in patients with impaired VFM after thyroid surgery. This analysis showed that, compared to the R Group, the U Group (particularly the U2 Group) had larger proportions of patients with EMG decrease >90% in the injured RLN and patients with thermal injury as the RLN injury mechanism (**Tables 1**, **2**). Voice parameter changes were much larger in the U Group compared to the R Group, but the change did not reach statistical significance (**Figure 2**). A large pitch range decrease in period-II is a prognostic indicator of moderate/severe long-term postoperative subjective voice impairment (**Figure 3**). Therefore, surgeons should make every effort to avoid severe type RLN injury during thyroid surgery and should utilize voice analysis to enable early identification of patients who may be experiencing distress and low satisfaction caused by poor surgical outcomes and to enable early initiation of intervention therapy to maintain quality of life in these patients.

The EMG data in revealed four interesting findings regarding injury mechanisms and postoperative VFM outcomes (**Table 1**). 1) No patient with EMG <50% decrease had postoperative asymmetric VFM. This finding indicates that preserving RLN function during surgery is the key factor in VFM preservation after surgery. Accurately recording all data for asymmetric VFM before surgery is also essential for avoiding misjudgment of vocal cord function and potential legal liability (20). 2) Patients with EMG 50-90% decrease in the injured RLN may not achieve total recovery from asymmetric VFM. Using continuous IONM can minimize complications by avoiding surgical procedures that can cause mechanical injury (21). Continuous IONM prevents most mechanical injuries with visually intact RLN, thereby enabling modification of injury-causing surgical procedures in 80% of cases (22). The advancement of continuous IONM technology can make a breakthrough in VFM evaluation after thyroidectomy (23). 3) All patients with thermal RLN injury in this study had unrecovered VFM (U Group). RLN thermal injury is difficult to be detected visually and the risk of permanent palsy is high, surgeons should maintain vigilance in modern thyroid surgery that increases the use of energy-based devices (24). Compared to relatively mild mechanical injuries, thermal injuries sustained during surgery may require earlier voice intervention (25) performed under comprehensive postoperative laryngofiberscopy and voice analysis. 4) The R Group and the U Group in this study did not significantly differ in Type 1 and Type 2 mechanical injury. In severe Type 2 (global) mechanical injury, VFM may have chance to be unrecovered.

In current study, patients in U group had higher proportion of malignant pathologic report than patients in R group (73.2% vs. 42.9%). For malignant disease, surgeons tend to dissect a longer segment in proximal end of RLN and perform central neck dissection; and tend to dissect the most distal end of RLN near laryngeal entry to decrease the tissue remnant in malignant disease. According to a large international registry database study with 1,000 RLNs at risks enrolled, the abnormal RLN trajectory (23%) was higher than surgeon expected, and 34% of RLN with loss of signal following an abnormal trajectory, for instance, fixed/splayed/entrapped RLN at the ligament of Berry, extensive RLN dissection, cases of cancer invasion or when lateral lymph node dissection (26). In this manner, the risk of RLN mechanical and thermal injury rate can be higher in malignant disease.

Some of the patients who revealed asymmetric VFM under laryngofiberscopy still had good subjective voice performance, which has been mentioned previously (27, 28). Reiter et al. (29) reported that voice parameters did not significantly differ between patients with and patients without recovery from vocal cord paralysis at the end of a 12-month follow up. In the current study, the R Group and the U Group did not significantly differ in objective or subjective voice parameters in all follow-up periods. However, U Group had much larger impairments compared to R Group, especially in ΔFmax, ΔPR, ΔJitter, ΔIVST-T, ΔIVST-V, and ΔIVST-S in **Figure 2**. The likely explanation for the lack of significant between-group differences was the large within-group differences in subjective voice outcomes. As **Table 2** indicates, the U1 and U2 Groups significantly differed in the incidence of severe RLN injury (i.e., EMG >90% decrease in injured RLN) and in RLN injury mechanism (thermal injury). Therefore, thyroid surgeons should make every effort to avoid severe type RLN injury. In the literature, several technical maneuvers had been reported for rescuing RLN function, including intravenous steroid (30), nimodipine (31), cold dextrose solution irrigation (32), etc., but the effectiveness of these methods remain uncertain. Besides of RLN factors, sufficient neuromuscular compensation plays an important role in reducing voice impairment after thyroid surgery (15, 33). Therefore, for good long-term postoperative voice outcomes after thyroid surgery, surgeons should prioritize restoration of neuromuscular compensation. Achieving the maximum neuromuscular compensation requires a multidisciplinary approach, especially with the participation of a speech-language pathologist to perform speech and dysphagia therapy (34). Other advanced interventions such as augmentation laryngoplasty and nerve reinnervation also have important rehabilitative roles in patients with voice recovery failure (35, 36).

In many institutions, the clinical practice environment may not include a thyroid surgeon with a laryngology background and may not have well-established procedures for routine cooperation between surgeons and speech-language pathologists (34). Under these conditions, compensation for voice impairment after thyroid surgery may not occur until long after surgery, which complicates detection of complications and other outcomes. Inadequate personnel and practices such as these may also mislead surgeons to believe that follow-up laryngofiberscopy and voice analysis are unnecessary. Early identification of voice impairment caused by VFM asymmetry can reduce the number of patient consultations needed to address psychological distress caused by poor voice outcomes and related concerns. It is worth mentioning that the voice impairment related to external branch of superior laryngeal nerve (EBSLN) injury is less significant than that of iatrogenic RLN injury. However, the stable EBSLN IONM (37) and careful analysis of high-pitched voice change (16) after thyroidectomy will largely improve life quality of the patients.

In terms of screening and interpreting voice outcomes, subjective voice analysis using IVST still has advantages over objective voice analysis. First, since a normal range of objective voice parameters has not been established, objective voice analysis requires time-consuming calculations of voice parameter changes (Δ) in individual patients whereas subjective voice analysis avoids this limitation. Second, for classifying patients with a wide range of long-term postoperative subjective voice impairments, e.g., the U Group in this study, IVST-T is simpler and more acceptable than objective voice parameters. Nevertheless, comprehensive objective voice analysis is still preferable for collecting and analyzing detailed voice data and for using these data as prognostic indicators of subjective voice impairment (**Figure 3**). Third, in patients with vocal cord paralysis, change in voice quality and loudness are among the most common complaints (38). Since objective voice recording method was used in the current study, loudness adjustments were made during recording, which limited objective assessments of loudness changes. Such changes could only be represented by IVST scores.

Several limitations of this study should be mentioned. First, demographic characteristics that may affect preoperative objective voice parameters differed between R Group and U Group. Future research may investigate novel preoperative objective voice parameters with less demographic variation and define “normal” ranges for such parameters. Second, no system for classifying nerve reinnervation or grafting has been established. Therefore, future works should consider how to account for the wide variation in nerve function outcomes after these procedures. Last, a longer observation time (e.g., 1 year) may be needed to observe voice compensation processes. However, the current data were sufficient for initial identification of trends in voice parameter changes after thyroidectomy. A future long-term post-thyroidectomy voice study should discuss and compare voice interventions (e.g., voice therapy, augmentation laryngoplasty, and medialization thyroplasty) and their effects.

## Conclusion

This study is the first to evaluate correlations between IONM findings and voice outcomes in patients with VFM impairment after thyroid surgery. In addition to assessing EMG status and RLN injury mechanisms, thyroid surgeons should routinely include laryngofiberscopy and subjective/objective voice analyses as standard tools for evaluation and diagnosis before and after thyroid surgery. Thyroid surgeons should also make every effort to avoid severe type (EMG >90% decrease, thermal-related) RLN injury, which is associated with a high risk of unrecovered VFM and with a high risk of moderate-to-severe long-term postoperative subjective voice impairment. Using objective voice parameters (e.g., pitch range) as prognostic indicators only enables surgeons to earlier identify patients who have low voice satisfaction after surgery, it also enables early implementation of intervention therapy (e.g., speech therapy, augmentation laryngoplasty, and nerve reinnervation), which can maximize neuromuscular compensation in these patients and maintain their quality of life.

## Data Availability Statement

The original contributions presented in the study are included in the article/**Supplementary Material**. Further inquiries can be directed to the corresponding author.

## Ethics Statement

Ethical approval of this study was obtained from the Kaohsiung Medical University Hospital Institutional Review Board (KMUHIRB-E(I)-20200359). The patients/participants provided their written informed consent to participate in this study.

## Author Contributions

Supervision – F-YC, C-WW, K-WL, and S-HL. Materials – T-YH, W-HY, F-YC, and C-WW. Data Collection and Processing – T-YH, W-HY, and S-HL. Analysis and Interpretation- T-YH, S-CF, A-ST, and S-HL. Literature Search - T-YH, W-HY, Y-CL, H-YT, and S-HL. Writing Manuscript – All authors. All authors have read and agreed to the published version of the manuscript.

## Funding

This study was supported by grants from Kaohsiung Medical University Hospital, Kaohsiung Medical University (KMUH109-9M44), Kaohsiung Municipal Siaogang Hospital/Kaohsiung Medical University Research Center grants (KMHK-DK(C)110009, I-109-04, H-109-05, I-108-02), and Ministry of Science and Technology (MOST 108-2628-B-037-006, MOST 109-2628-B-037-014, MOST 110-2636-B-009-008, MOST 110-2314-B-037-104-MY2, MOST 110-2314-B-037-120), Taiwan.

## Conflict of Interest

The authors declare that the research was conducted in the absence of any commercial or financial relationships that could be construed as a potential conflict of interest.

## Publisher’s Note

All claims expressed in this article are solely those of the authors and do not necessarily represent those of their affiliated organizations, or those of the publisher, the editors and the reviewers. Any product that may be evaluated in this article, or claim that may be made by its manufacturer, is not guaranteed or endorsed by the publisher.
