# Supplementary material for: Prognostic Indicators of Non-Transection Nerve Injury and Vocal Fold Motion Impairment After Thyroid Surgery – Correlation Between Intraoperative Neuromonitoring Findings and Perioperative Voice Parameters
Source: Front Endocrinol (Lausanne). 2021 Nov 30;12:755231. doi: 10.3389/fendo.2021.755231 (PMC8669766; doi:10.3389/fendo.2021.755231)
Supplement: Supplementary file 1 [file Table_1.docx]

**Supplemental Table 1**. Index of Voice and Swallowing Handicap of Thyroidectomy (IVST)

| **Questions** | **Never**  **(0 point)** | **Sometimes**  **(1 point)** | **Always**  **(2 points)** |
| --- | --- | --- | --- |
| Voice domain |  |  |  |
| 1. My overall voice quality is abnormal. | 0 | 1 | 2 |
| 1. My voice difficulties restrict personal and social life. | 0 | 1 | 2 |
| 1. I feel my voice is hoarse. | 0 | 1 | 2 |
| 1. I feel as though I have to strain to produce voice. | 0 | 1 | 2 |
| 1. The sound of my voice varies throughout the day. | 0 | 1 | 2 |
| 1. I find it difficult to make a high-pitched voice. | 0 | 1 | 2 |
| 1. I find it difficult to make a low-pitched voice. | 0 | 1 | 2 |
|  | **IVST-V = ______ (Range from 0-14)** | | |
| Swallowing domain |  | | |
| 1. I feel strained when I speak or swallow. | 0 | 1 | 2 |
| 1. I choke when I drink (water or tea). | 0 | 1 | 2 |
| 1. I choke when I eat. | 0 | 1 | 2 |
|  | **IVST-S = ______ (Range from 0-6)** | | |
| Total score | **IVST-T = ______ (Range from 0-20)** | | |

**Supplemental Table 2.** Comparison of voice parameter and voice parameter changes (Δ) of Recovered VFM Group (R Group) and Unrecovered VFM Group (U Group)

| Period | Parameters | R Group | U Group | p value | Parameter  changes(Δ) | R Group | U Group | p value |
| --- | --- | --- | --- | --- | --- | --- | --- | --- |
| I  II  III  IV | Fmin  (Hz) | 127.9±33.5  122.3±37.3  120.5±36.4  119.7±29.5 | 126.2±31.4  135.9±33.7  125.9±41.1  124.3±20.6 | 0.833  0.117  0.609  0.620 | ΔFmin  (%) | -  -3.4±24.4  -1.1±28.7  -1.5±20.3 | -  4.3±27.8  -0.8±22.9  -9.3±16.5 | -  0.198  0.967  0.243 |
| I  II  III  IV | Fmax  (Hz) | 575.6±208.4  404.6±162.7  486.0±214.9  518.6±214.6 | 710.2±245.0  443.5±167.3  497.0±191.8  602.5±192.5 | **0.018***  0.325  0.850  0.248 | ΔFmax  (%) | -  -27.1±27.5  -11.0±32.4  -3.2±36.5 | -  -30.5±33.4  -23.4±30.1  -13.8±46.6 | -  0.627  0.105  0.515 |
| I  II  III  IV | PR  (Semitone) | 25.5±6.8  20.4±7.2  22.9±7.9  24.6±6.2 | 29.4±6.8  20.5±6.8  23.4±8.8  26.4±6.9 | **0.028***  0.967  0.842  0.413 | ΔPR  (%) | -  -15.9±34.9  -6.6±37.6  1.7±36.3 | -  -26.3±23.9  -17.3±29.8  -6.7±28.9 | -  0.129  0.186  0.467 |
| I  II  III  IV | Mean F0  (Hz) | 186.8±39.5  169.9±48.6  175.9±44.2  188.3±44.2 | 204.4 ±43.2  191.7±45.8  198.4±44.4  194.6±45.2 | 0.091  0.057  0.073  0.682 | ΔMean F0  (%) | -  -8.9±17.9  -5.2±16.3  -1.4±12.1 | -  -3.8±13.8  0.6±16.1  -13.2±19.2 | -  0.164  0.145  0.081 |
| I  II  III  IV | Jitter  (%) | 1.7±1.0  2.3±1.8  2.2±1.5  1.8±1.3 | 1.3±0.9  2.0±1.1  2.0±1.1  1.9±1.4 | 0.077  0.384  0.618  0.785 | ΔJitter  (%) | -  64.7±126.2  54.6±135.6  30.5±90.1 | -  125.9±205.0  131.3±208.8  131.8±221.3 | -  0.124  0.086  0.170 |
| I  II  III  IV | Shimmer  (%) | 3.6±2.1  4.4±4.6  4.6±4.8  3.6±2.2 | 3.2±1.8  3.3±1.9  3.6±2.7  4.1±2.0 | 0.368  0.243  0.399  0.539 | ΔShimmer  (%) | -  33.4±109.5  29.6±114.9  34.8±72.3 | -  24.5±94.9  39.8±117.8  53.3±77.3 | -  0.707  0.718  0.512 |
| I  II  III  IV | NHR  (value) | 0.1±0.0  0.1±0.0  0.1±0.0  0.1±0.0 | 0.1±0.0  0.1±0.0  0.1±0.0  0.1±0.0 | 0.679  0.071  0.950  0.809 | ΔNHR  (%) | -  13.8±35.6  5.1±35.6  14.2±27.0 | -  1.5±31.4  4.4±41.1  12.3±29.6 | -  0.112  0.927  0.858 |
| I  II  III  IV | IVST-T (score) | 0.6±1.3  4.3±3.4  3.5±3.9  3.3±3.7 | 0.4±1.2  7.5±5.1  7.5±5.7  7.6±6.4 | 0.737  0.113  0.085  0.145 | ΔIVST-T (score) | -  3.7±2.7  3.1±3.3  2.7±3.3 | -  6.5±6.7  7.3±6.2  7.1±6.8 | -  0.204  0.062  0.140 |
| I  II  III  IV | IVST-V (score) | 0.4±1.2  3.1±2.6  2.9±3.3  2.4±2.8 | 0.2±0.6  5.6±3.8  5.8±4.1  5.6±4.8 | 0.660  0.105  0.097  0.135 | ΔIVST-V (score) | -  2.7±2.2  2.5±3.0  1.7±2.9 | -  4.9±5.1  5.7±4.4  5.3±5.0 | -  0.197  0.063  0.112 |
| I  II  III  IV | IVST-S (score) | 0.2±0.6  1.2±1.0  0.6±1.0  0.9±1.7 | 0.2±0.6  1.9±1.5  1.7±0.7  2.0±2.2 | 1.000  0.230  0.135  0.346 | ΔIVST-S (score) | -  1.0±1.2  0.6±0.9  1.0±1.8 | -  1.6±1.7  1.6±1.9  1.8±2.3 | -  0.370  0.107  0.474 |

VFM = vocal fold motion

Period I = Preoperative period (within 2 months before surgery); Period II = Perioperative period (median duration of 3 days; range of 1-7 days); Period III = Short-term postoperative period (median duration of 12 days; range of 7-30 days); Period IV = Long-term postoperative period (median duration of 40 days, range of 30-90 days)

* p value <0.05, showed significant difference
